# Transplantation of embryonic spleen tissue reveals a role for adult non-lymphoid cells in initiating lymphoid tissue organization

**DOI:** 10.1002/eji.200838724

**Published:** 2009-01

**Authors:** Stephanie H Glanville, Vasileios Bekiaris, Eric J Jenkinson, Peter J L Lane, Graham Anderson, David R Withers

**Affiliations:** MRC Centre for Immune Regulation, Institute for Biomedical Research, Birmingham Medical SchoolBirmingham, UK

**Keywords:** Animal models, Lymphoid organs, Spleen, Transplantation

## Abstract

In this report we describe a transplantation system where embryonic spleens are grafted into adult hosts. This model can be used to analyze the cellular and molecular requirements for the development and organization of splenic microenvironments. Whole embryonic day 15 (ED15) spleens, grafted under the kidney capsule of adult mice, were colonized by host-derived lymphocytes and DC and developed normal splenic architecture. Grafts were also able to form germinal centers in response to T-dependent antigen. Using this system we demonstrated that adult host-derived lymphotoxin (LT) α was sufficient for the development of ED15 LTα^−/−^ grafts. Grafting of ED15 LTα^−/−^ spleens into RAG^−/−^ hosts followed by transfer of LT α^−/−^ splenocytes revealed no requirement for lymphocyte-derived LT α in the induction of CCL21 or the development of T-zone stroma. These data suggest that interactions between adult lymphoid-tissue inducer-like cells and embryonic stromal cells initiated T-zone development. Furthermore, adult lymphoid tissue inducer-like cells were shown to develop from bone marrow-derived progenitors. The model described here demonstrates a method of transferring whole splenic microenvironments and dissecting the stromal and hematopoietic signals involved in spleen development and organization.

## Introduction

The generation of efficient immune responses to T-dependent Ag requires organization and segregation of the B- and T-cell populations within secondary lymphoid tissue 1–3. This segregation is thought to be mediated by site-specific expression of homeostatic chemokines; CCL19 and CCL21 that bind CCR7, expressed at high levels by T cells, and CXCL13 that binds CXCR5, expressed mainly by B cells 4. Expression of these chemokines by distinct stromal cell populations controls the movement of B and T cells within secondary lymphoid tissue 4–6. Chemokines are thought to be disseminated by a network of stromal cells and collagen fibers termed the conduit system 7 and it is this framework that guides the movement of cells within the tissue 8.

The development and organization of secondary lymphoid tissue is dependent upon signals downstream of the β-receptor lymphotoxin (LT) since in the absence of either this or its ligand, LTα1β2, LN and Peyer's patches (PP) are absent and although lymphocytes are present in the spleen, B- and T-cell compartmentalization is lost 9, 10. A role for CD30 has also been identified in the segregation of splenic B and T cells, although the mechanism remains unclear 11. During both LN and PP development, lymphoid tissue inducer (LTi) cells, a population of CD45^+^CD3^−^CD11c^−^B220^−^CD4^+^IL-7Rα^+^ cells that express LTα1β2 and α4β1 12–17 cluster with VCAM-1^+^ICAM-1^+^ stromal cells that express LTβ-R. Interactions between these cell types results in the engagement of the LTβ-R on the stromal cells, expression of CCL19, CCL21 and CXCL13, and the subsequent recruitment of lymphocytes to the site 15, 18, 19. Since LTα1β2 is expressed by B cells, the influx of lymphocytes greatly amplifies expression of the homeostatic chemokines, driving further recruitment. A novel population of CD45^+^CD4^+^CD11c^+^ cells that express the receptor tyrosine kinase RET has also been shown to be involved in early PP development 20. Engagement of the RET ligand, ARTN, expressed by gut stromal cells is proposed as a mechanism through which LTi are recruited to the sites of PP formation. Whether these cells and the RET/ARTN signaling pathway are involved in the formation of other secondary lymphoid tissue is unclear.

Recently, a role for LTi in the development of the splenic white pulp was identified, with clusters of LTi and VCAM-1^+^ stromal cells detected within the embryonic spleen, as described for LN and PP 21. Although B-cell-derived LTα1β2 has been shown to induce expression of homeostatic chemokines in the spleen 22, LTi clustering in the embryonic spleen occurs several days prior to lymphocyte colonization of the tissue and has been shown to induce expression of VCAM-1 in an LTα-dependent manner. Furthermore, through transfer of LTα^−/−^ splenocytes into RAG^−/−^ mice, it was demonstrated that B-cell-derived LTα1β2 is not essential for expression of CCL21 or the T-zone stromal marker podoplanin 21. These data are consistent with LTi delivering an early LTα1β2 signal during spleen development, however there seems to be redundancy in the source of LTα1β2, since mice deficient in either retinoic acid orphan receptor (ROR)γ or inhibitor of DNA binding 2 (ID2) lack LTi and although LN and PP are absent, there is normal splenic architecture 23–25.

Cells with a very similar phenotype to LTi and thus termed LTi-like cells can be detected within adult secondary lymphoid tissue 26, 27. Unlike LTi in the embryo, these cells constitutively express OX40L and CD30L, through which they can mediate memory T-cell survival 26–28. Although transfer of LTi-like cells into adult LTα^−/−^ mice caused the expression of CCL21 and improved B- and T-cell segregation 29, it is not clear whether LTi-like cells are able to initiate secondary lymphoid tissue development or mediate the organization of B and T cells in developing tissue. In this respect, the functional relationship between the embryonic LTi and adult LTi-like cells remains unknown.

In this report we describe a transplantation system in which whole embryonic spleen tissue is grafted under the kidney capsule of adult allotype marked mice. In this model, the embryonic tissue develops normal splenic architecture and function. Using this system, we demonstrate that there is no requirement for LTα in spleen development prior to embryonic day 15 (ED15), since ED15 LTα^−/−^ grafts developed normally in WT hosts. This demonstrated that LTα expressed by host-derived cells was sufficient for development. Adult LTi-like cells from the host were detected within the grafted tissue and in the absence of lymphocyte-derived LTα, expression of CCL21 and podoplanin was induced in developing white pulp areas. In light of current evidence regarding secondary lymphoid organ development we propose that the cell type mediating this organization is adult LTi-like cells and further show that these cells can be generated from adult bone marrow progenitors.

## Results

### Development of functional spleen tissue from ED15 spleen grafts

**Figure 1:**
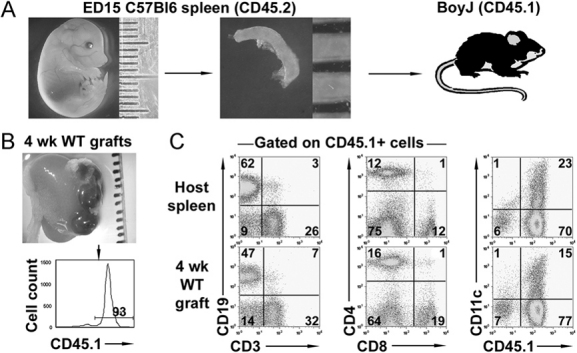
Normal development of WT embryonic spleen grafts. Embryonic spleen isolated from C57BL6 (CD45.2) embryos was grafted under the kidney capsule of adult BoyJ (CD45.1) mice and then analyzed 4 wks later. (A) Whole ED15 embryo and isolated spleen. (B) Embryonic spleen grafts after 4 wk contained mostly CD45.1^+^ hematopoietic cells. (C) Host- and WT-graft-derived cells analyzed for expression of CD19 *versus* CD3 and CD4 *versus* CD8 amongst CD45.1^+^ cells and CD11c *versus* CD45.1.

**Figure 2:**
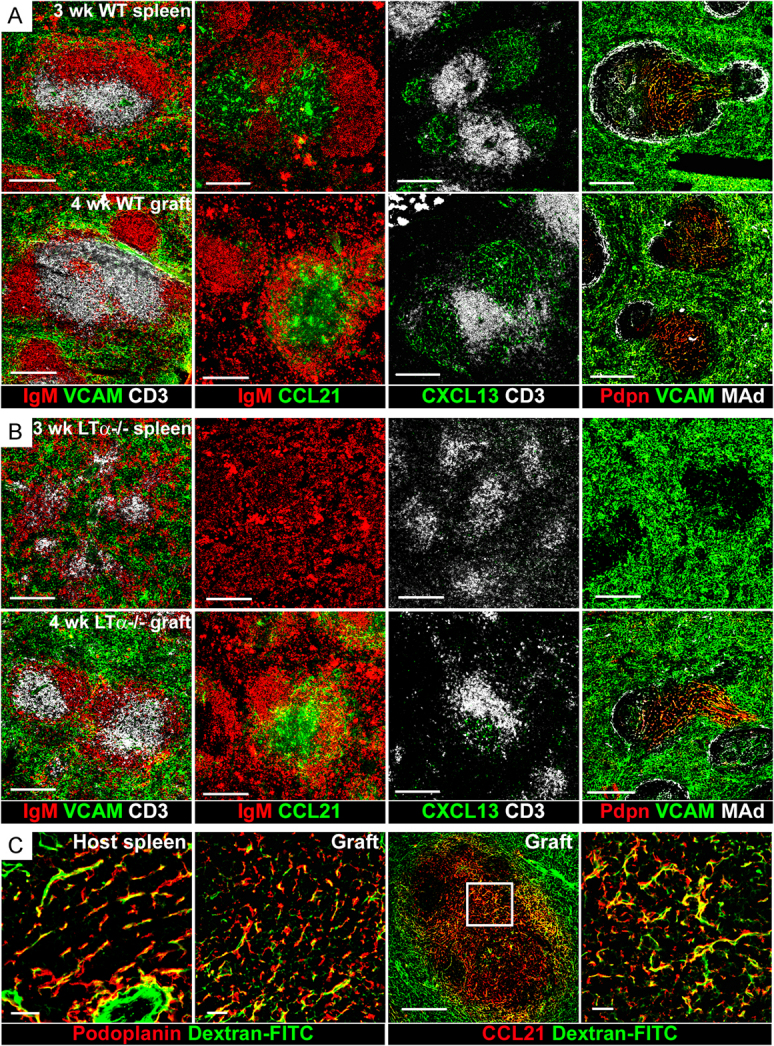
Normal splenic architecture in WT and LTα^−/−^ embryonic spleen grafts. Embryonic spleen isolated from WT or LTα^−/−^ (C57BL6 background, CD45.2) embryos was grafted under the kidney capsule of adult BoyJ (CD45.1) mice and then analyzed 4 wk later. (A) Expression of either IgM or podoplanin (red), VCAM-1, CCL21, CXCL13 (green) and CD3 or MAdCAM-1 (white) in sections of 3 wk WT (upper panels) or grafted WT spleen after 4 wk (lower panels). (B) Expression of either IgM or podoplanin (red), VCAM-1, CCL21, CXCL13 (green) and CD3 or MAdCAM-1 (white) in sections of 3 wk LTα^−/−^ (upper panels) or grafted LTα^−/−^ spleen after 4 wk (lower panels). (C) Visualization of dextran-FITC (green) with either podoplanin or CCL21 expression (red) in host- and WT-grafted spleen. Scale bars represent 200 μm. Data are representative of at least three separate experiments.

To investigate the ability of cells within the adult mouse to influence the development of embryonic tissue, an embryonic spleen-grafting model was established, similar to those used to study thymus and LN development 30, 31. Developing spleen tissue was isolated at ED15, the time when the initial LTi interactions with VCAM-1^+^ stroma are thought to occur and prior to lymphocyte colonization. Whole ED15 spleens, isolated from C57BL6 embryos (CD45.2^+^), were grafted under the kidney capsule of adult CD45.1^+^ WT mice (Fig. 1A). The grafts were removed 4 wk later, by which time they had developed a ‘splenic’ morphology with formation of red and white pulp areas (Fig. 1B). Hematopoietic cells within the graft were mostly of host origin including B cells, both CD4^+^ and CD8^+^ T cells and CD11c^+^ DC (Fig. 1B and C). Importantly, these cells were distributed into T-zone areas with associated B-cell follicles, surrounded by an IgM^+^IgD^−^ marginal zone (Fig. 2A and Supporting Information Fig. 1). Consistent with this organization, expression of CCL21 was detected within the T zone and CXCL13 was expressed within the follicular areas. Furthermore, the white pulp stromal cells of the grafts also developed normally with expression of podoplanin restricted to the T zone and MAdCAM-1 to the marginal sinus (Fig. 2A). A few graft-derived (CD45.2^+^) cells were detected within the grafts, most of which expressed the macrophage marker F4/80 (data not shown). Since the ED15 spleen contains both CD11b^+^Gr-1^−^ and CD11b^+^Gr-1^+^ cells (data not shown), this is consistent with the persistence of these macrophage-like cells.

To confirm that the conduit system had developed normally within the grafts, grafted mice were injected with the fluorescent tracer FITC-dextran (10 kDa). The distribution of the FITC-dextran in the grafts was comparable to that in the host spleen, with cells expressing podoplanin ensheathing FITC^+^ channels (Fig. 2C). Also, expression of CCL21 appeared to co-localize with the injected FITC-dextran indicating the formation of a functional conduit in which chemokines are disseminated. Grafted mice were also immunized with sheep red blood cells and after 10 days formed clear peanut agglutinin-positive GC structures demonstrating that the white pulp areas in the graft could respond to T-dependent Ag (Supporting Information Fig. 2). Therefore, the grafting of whole ED15 spleens resulted in the development of splenic tissue containing mostly host-derived CD45^+^ cells. These cells were located within organized white pulp areas, which supported T-dependent immune responses. This system therefore provides an ideal model to further investigate aspects of spleen development and organization.

### Rescue of embryonic LTα^−/−^ spleens by grafting into WT mice

The ED15 spleen appeared to contain all the embryonic-derived factors required for normal splenic development. Whether LTα signals are required before ED15 in splenic development is unknown and recently the initial stages of LN formation were shown to be LTα-independent 30. To investigate this, ED15 LTα^−/−^ spleens were grafted into WT host mice and after 4 wk the grafts contained CD45^+^ cells almost exclusively of host origin, including the major lymphocyte subsets and DC (data not shown). Unlike a LTα^−/−^ spleen, the grafted LTα^−/−^ tissue contained organized white pulp areas with clear segregation of B and T cells and expression of CCL21 and CXCL13 within the T zone and B-cell follicles, respectively (Fig. 2B). A marginal zone of IgM^+^IgD^−^ B cells was also formed (Supporting Information Fig. 1). Furthermore, expression of podoplanin, undetectable in the absence of LTα 22, was detected in the T zone and MAdCAM-1 expression was detected at the marginal sinus (Fig. 2B). The grafted LTα^−/−^ spleens were also able to form GC in response to immunization with sheep red blood cells (data not shown). This organization was dependent upon host cells expressing LTα since grafting of LTα^−/−^ embryonic spleens into LTα^−/−^ adult mice resulted in disorganized lymphocyte aggregates with no detectable homeostatic chemokines (Supporting Information Fig. 3).

**Figure 3:**
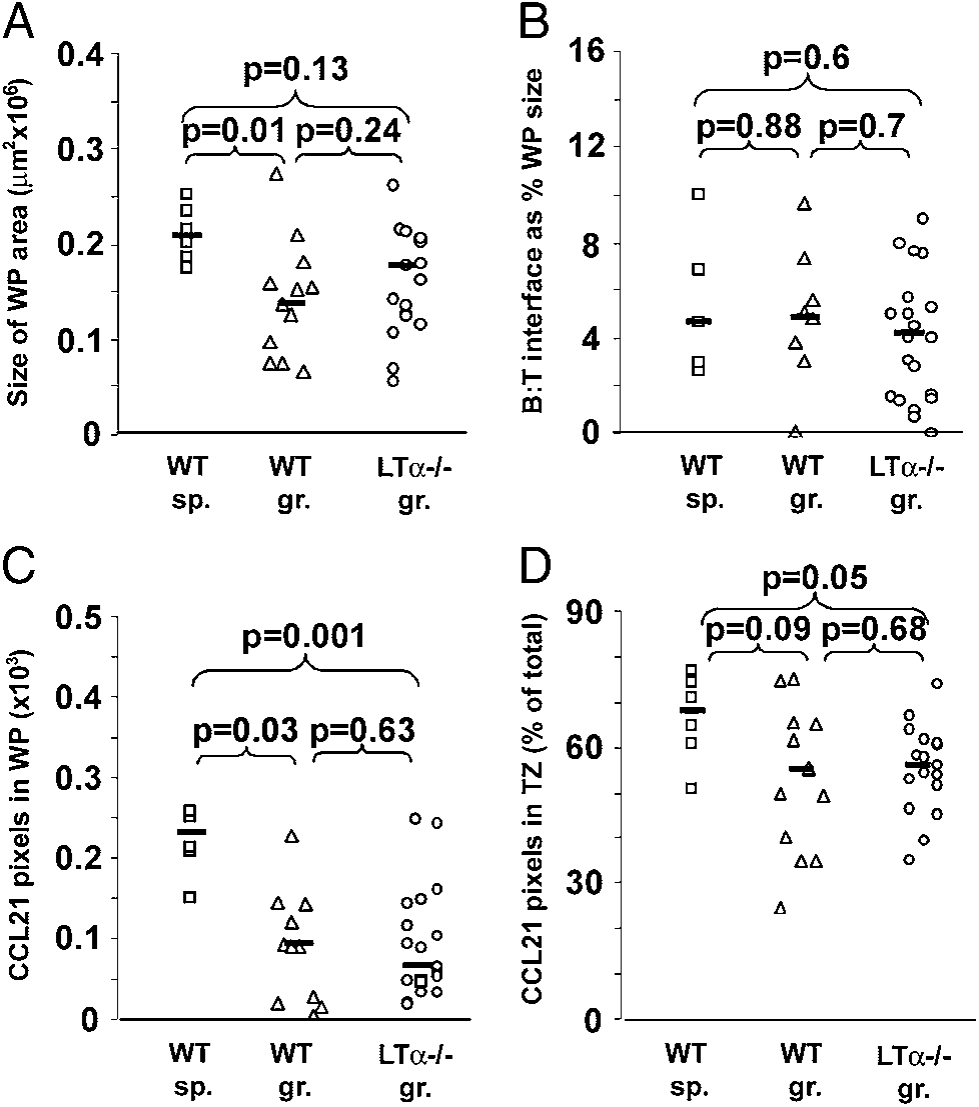
Quantitative analysis of immunofluorescent data from grafted tissue. To confirm normal development of LTα^−/−^ grafts, the immunofluorescent data were quantitatively analyzed. Comparisons were made between 3 wk WT spleens and 4 wk WT and LTα^−/−^ splenic grafts. (A) Size (μm^2^) of white pulp areas. (B) Area of the B/T interface as a percentage of the total white pulp area. (C) Total number of CCL21^+^ pixels in white pulp. (D) CCL21 pixel number within the T zone as a percentage of the total pixel number. *p* values show results of non-parametrical Mann–Whitney *U*-test, bars represent median. Each symbol represents one white pulp area analyzed, graphs were compiled from at least three separate experiments.

The size of white pulp areas in either WT or LTα^−/−^ grafts was not significantly different (*p*=0.24), although they were smaller than those within normal spleen tissue of comparable age (WT grafts *p*=0.01, LTα^−/−^ grafts *p*=0.13, Fig. 3A). Importantly, analysis of the size of the interface between B and T cells, indicative of the efficiency of B- and T-cell segregation, showed that the grafted white pulp areas (WT or LTα^−/−^) were as well organized as normal splenic tissue (WT grafts *p*=0.88, LTα^−/−^ grafts *p*=0.6, Fig. 3B). There was also no significant difference between WT and LTα^−/−^ grafts (*p*=0.7). Finally, analysis of the CCL21 expression again revealed no significant difference between the WT and LTα^−/−^ grafts (*p*=0.63), although the amount of CCL21 was significantly reduced compared to normal spleen (WT grafts *p*=0.03, LTα^−/−^ grafts *p*=0.001, Fig. 3C). CCL21 expression was also less restricted to the T zone although this difference was not statistically significant (WT grafts *p*=0.09, LTα^−/−^ grafts *p*=0.05, Fig. 3D). This latter observation may reflect differences in the vascularization of the grafts and host spleen and there was strong correlation between WT and LTα^−/−^ grafts (*p*=0.68). In summary, ED15 LTα^−/−^ grafts developed organized white pulp areas comparable to WT ED15 spleen grafts.

### Host adult LTi-like cells traffic to the grafted tissue

**Figure 4:**
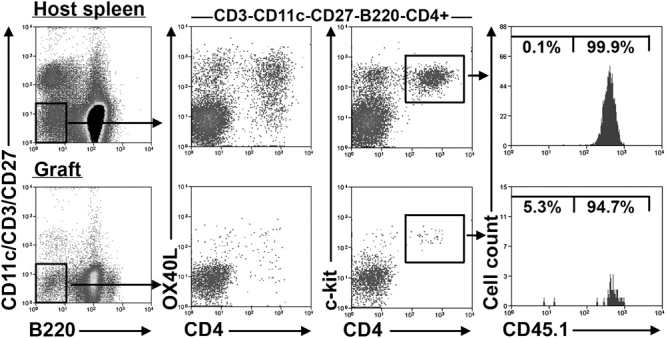
Recruitment of host LTi-like cells to the splenic grafts. To investigate whether host LTi-like cells were present within the embryonic spleen grafts, ED15 C57BL6 spleens (CD45.2) were grafted under the kidney capsule of adult CD3ɛ (CD45.1) mice and then analyzed 4 wk later by flow cytometry. Expression of CD4 with either c-kit or OX40L amongst CD11c^−^B220^−^ cells in the host spleen and grafts; CD3^−^CD11c^−^CD27^−^B220^−^CD4^+^c-kit^+^ cells further analyzed for expression of CD45.1. Numbers show percentage positive cells within gate. Data are representative of three grafted mice.

The recovery of normal splenic architecture in the ED15 LTα^−/−^ spleens was dependent upon the trafficking of host cells expressing LTα to the graft. Although B cells express LTα1β2, LTi colonize the spleen several days before lymphocytes and provide early signals to the developing stroma that form the T zone 21. To investigate whether LTi-like cells were present in the grafted tissue, ED15 spleens were grafted into CD3ɛ mice, which lack T cells enabling the easier identification of CD4^+^CD3^−^ cells. After 4 wk, grafted and host splenic tissue was analyzed for the presence of graft- (CD45.2) and host-derived (CD45.1) cells. Within the grafts a population of CD3^−^CD11c^−^CD27^−^B220^−^CD4^+^ cells expressing c-kit and OX40L was detected (Fig. 4). This population was composed of mostly CD45.1^+^ cells demonstrating that host LTi-like cells were indeed present in the grafts. There was no reconstitution of the host (CD3ɛ) thymus in grafted mice and no T cells were detected in either the host or grafted spleen (data not shown). The detection of CD45.1^−^ LTi indicates that LTi present in the grafted tissue can persist for at least 4 wk.

### Adult non-lymphoid cells induce T-zone development

**Figure 5:**
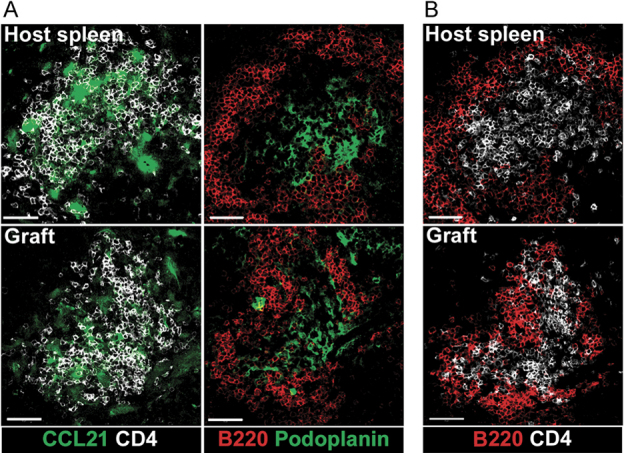
Adult LTi-like cells are sufficient for organization of developing white pulp areas. To test whether the adult LTi-like cells detected within the grafts were sufficient for the development of organized white pulp areas, ED15 LTα^−/−^ spleens were grafted into adult RAG^−/−^ mice; after 4 wk LTα^−/−^ splenocytes were injected i.v. and then 4 wk later the host spleen and grafts were analyzed by immunofluorescence. (A) CD4 (white) and CCL21 (green) and B220 (red) and podoplanin (green) in host spleen (upper panels) and grafts (lower panels). (B) Expression of B220 (red) and CD4 (white) in host spleen (upper panels) and grafts (lower panels). Scale bar represents 20 μm. Pictures are representative of two separate experiments.

Since the grafted tissue contained both graft- and host-derived LTi expressing LTα1β2, as well as B cells, it was unclear whether adult LTi-like cells contributed to the organization of the developing white pulp areas. To test whether the grafts required adult lymphocyte-derived LTα for induction of white pulp development, ED15 LTα^−/−^ spleens were grafted into RAG^−/−^ mice, and after 2 wk the grafted mice were injected with LTα^−/−^ splenocytes. Thus the only cells expressing LTα within these mice were present within the RAG^−/−^ adult. The grafted tissue was then analyzed 3 wk later and compared with the host spleen. The grafted tissue contained areas where expression of CCL21 co-localized with clusters of T cells within the grafts (Fig. 5A). Furthermore, expression of podoplanin in these areas indicated the development of T-zone stroma (Fig. 5A). Surrounding these rudimentary T zones were rings of B cells (Fig. 5B), demonstrating that the lymphocyte populations were segregated into discrete compartments. Within the grafted tissue, but not the host spleen, aggregates of un-segregated B and T cells remained, consistent with a limited number of LT-expressing cells reaching the graft and subsequently the failure of some white pulp areas to organize (data not shown). These data show that a population of cells in the adult RAG^−/−^ mouse is able to colonize the embryonic grafts and induce the development of T-cell areas and the segregation of B and T cells.

### Evidence for the generation of LTi-like cells from the BM

**Figure 6:**
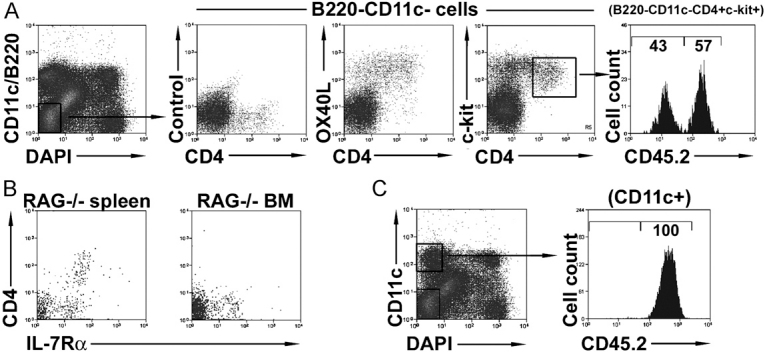
Generation of LTi-like cells in the adult from BM progenitors. To investigate whether LTi-like cells were generated from progenitors in the BM, CD3ɛ mice (CD45.1) were irradiated then reconstituted with RAG^−/−^ BM (CD45.2). (A) Expression of OX40L or c-kit *versus* CD4 amongst DAPI^−^CD11c^−^B220^−^ cells isolated from the spleen; B220^−^CD11c^−^CD4^+^c-kit^+^ cells further analyzed for expression of CD45.2. Numbers show percentage positive cells within gate. (B) Expression of CD4 *versus* IL-7Rα amongst lineage-negative cells isolated from RAG^−/−^ spleen or BM. (C) Expression of CD45.2 amongst CD11c^+^ cells isolated from the spleen of chimeric mice.

Whether adult LTi-like cells are post-natally generated from a BM progenitor population is unknown. LTi-like cells were not detected in the blood of CD3ɛ mice, in accordance with previous studies [29 and data not shown]. This would argue against the presence of a circulating population of these cells. To investigate whether a progenitor for the LTi-like cells existed within adult BM, CD3ɛ mice (CD45.1) were sub-lethally irradiated and then reconstituted with adult RAG^−/−^ (CD45.2) BM. After 4 wk, the spleens of the chimeric mice contained CD45.2^+^ LTi-like cells (Fig. 6A), demonstrating the presence of a progenitor population in the RAG^−/−^ BM. No LTi-like cells were detected within the initial RAG^−/−^ BM preparation (Fig. 6B), although a population of lineage-negative cells expressing IL-7Rα was detected. Importantly, host (CD45.2^−^)-derived LTi-like cells, but not DC, persist within the chimeric spleens (Fig. 6C), demonstrating that some LTi-like cells are resistant to irradiation.

## Discussion

We have previously described the presence of cells within adult secondary lymphoid tissue with a phenotype matching that of LTi 27. The transfer of these LTi-like cells into adult LTα^−/−^ mice improved B- and T-cell segregation and induced expression of CCL21 29. To further investigate the ability of adult cells to influence the development of secondary lymphoid tissue, a model in which whole embryonic spleens were grafted under the kidney capsule of adult mice was established. The grafting of WT ED15 spleens under the kidney capsule of WT mice resulted in tissue with a normal splenic morphology and architecture, containing host-derived lymphocyte and DC populations alongside mature stromal cell populations. Vondenhoff *et al.* 32 also use the grafting of embryonic spleen tissue under the kidney capsule where they show the development of B cells from fetal spleens. However, the use of RAG2^−/−^ common γ chain^−/−^ host mice in this study 32 may, given their lack of lymphocytes, affect the homeostatic proliferation of graft-derived cells. The normal development of LTα^−/−^ ED15 grafts demonstrated that the spleen does not require LTα signals prior to ED15 and, furthermore, LTα expressed by adult-derived cells was sufficient. The spleen-grafting technique described here has the advantage of being able to transplant the entire spleen microenvironment, minimizing tissue disruption and allowing cell–cell contact to take place in its natural environment. Furthermore, this model allows the dissection of both stromal and hematopoietic signals through the use of host and donor mice. In addition, the transplantation of spleen fragments in individuals undergoing splenectomy suggests that this model may be of clinical relevance 33.

Recently, the early stages of splenic white pulp formation were described as developing in a LTα1β2-independent manner with expression of LTα1β2 in the spleen limited to B cells at 4 days after birth 32. However, these data conflict with the clear LTα-dependent changes in splenic stromal cell populations that occur in the embryonic spleen, where clusters of LTi interact with VCAM-1^+^ stroma at the sites where T cells are recruited 21. Whilst the rescue of ED15 LTα^−/−^ spleen grafts also indicates a LT-independent stage to spleen development, current data indicate plasticity in the timing of LTα-dependent signals, particularly when the normal spleen development of RORγ^−/−^ mice is considered 24.

Given that the development of the LTα^−/−^ grafts was dependent upon the colonization of the tissue with host cells expressing LTα this model enabled an analysis of the ability of adult cells to influence the development of splenic white pulp. We therefore investigated whether development of the graft white pulp areas required adult lymphocyte-derived LTα. Detection of both CCL21 and podoplanin, corresponding with T-cell localization, demonstrated that cells within the RAG^−/−^ host could induce development of the T zone. The cells most likely to be responsible for this are the adult LTi-like cells that persist in RAG^−/−^ mice 21. It remains possible that other cells present in the RAG^−/−^ host, such as NK cells, could initiate CCL21 expression, however existing data argue against this; firstly, NK cells are not essential for splenic white pulp development since the spleens of CD3ɛ mice (T- and NK-cell deficient) develop a normal architecture, with a reduced T zone populated by DC. Secondly the localization of NK cells in the red pulp, unless specifically recruited through viral infection, also argues against a role in mediating white pulp development 34. Furthermore, LTi-like cells in adult mice have recently been shown to restore splenic architecture after viral infection 35.

Since LTi-like cells were not detected within the blood, it is unlikely that a circulatory population of these cells exists. The BM chimeras demonstrated that adult LTi-like cells in the spleen could be generated from BM-derived progenitors. This suggests that the adult LTi-like cells in the graft may also develop from BM-derived progenitors. Surprisingly, host-derived LTi-like cells, but not DC, were detected in the chimeras demonstrating that at least some LTi-like cells are resistant to irradiation. This observation has important implications for the interpretation of data derived from BM chimeras and may explain previous observations where LTα-deficient splenocytes were used to reconstitute irradiated RAG-1^−/−^ and WT mice 2, 36.

The identification of cells within the adult mouse that can initiate secondary lymphoid tissue development has clear implications for the generation of tertiary lymphoid tissue. Tertiary lymphoid tissue formation shares many similarities with that of secondary lymphoid tissue 37, 38. Ectopic expression of either LT 39, or the homeostatic chemokines CCL21 or CXCL13 40–43 in the pancreas resulted in the recruitment of T cells, B cells and DC and the subsequent generation of organized lymphoid aggregates with associated high endothelial venules at this site. While these models demonstrate that lymphocyte recruitment lies downstream of LT-signaling and subsequent chemokine expression, the critical early stages that result in the aberrant chemokine expression are missed. Since LTi are essential for the early stages of both LN and PP formation and given the strong similarities between secondary and tertiary lymphoid tissue development, it seems likely that LTi-like cells in the adult are involved early in the formation of tertiary lymphoid tissue. The varying levels of lymphocyte organization described for different tertiary lymphoid tissues 37 may reflect whether LTi-like, or other LT-expressing cells are recruited during the initial stages. We propose that during chronic inflammation either LTi-like cells or their progenitors may be recruited to the area where they interact with stromal cells, prompting the aberrant chemokine expression that recruits lymphocytes to the site. As in the development of secondary lymphoid tissue, the recruitment of B cells expressing LTα1β2 will amplify and expand the recruitment process driving the accumulation of lymphocytes and the formation of the lymphoid aggregate.

## Materials and methods

### Mice

All experiments were performed in accordance with UK laws and with the approval of the University of Birmingham ethics committee. WT (C57BL6, BoyJ and Balb/c), LTα^−/−^, RAG1^−/−^ (both on a C57BL6 background) and CD3ɛ-transgenic (CD3ɛ, BoyJ background) 44 mice were all bred and maintained in our animal facility. Spleens were removed from embryos on ED15 using a dissecting microscope and subsequently grafted under the kidney capsule of adult mice aged between 4 and 6 wk.

### Injection of tracer molecules and tissue preparation

Mice that had received kidney capsule grafts 4 wk previously were injected i.v. with 200 μL of 10 000 mW, lysine-fixable dextran-FITC (5 mg/mL) (Invitrogen, Paisley, UK). Mice were euthanized 5 min later, the grafts and host spleen removed, placed in 3% formalin for 3 h followed by washing in PBS and then 30% sucrose before freezing and sectioning.

### Immunization

Mice that had received kidney capsule grafts 4 wk previously were injected i.p. with 1×10^8^ sheep red blood cells (TCS Biosciences, Bucks, UK). Mice were euthanized after a further 10 days and the grafts and host spleen removed for analysis.

### Immunofluorescence

Six μm sections of tissue were cut and fixed in acetone. Antibodies used were anti-CD3ɛ (clone: 145-2C11, BD Pharmingen, Oxford, UK), anti-CD3ɛ biotin (145-2C11, eBioscience, San Diego, CA, USA), anti-CD45.2 FITC (104, eBioscience), anti-CCL21 or CXCL13 (R&D Systems, Minneapolis, MN, USA), anti-IgD FITC (Southern Biotech, Birmingham, AL, USA), anti-IgM Rhodamine Red (Jackson Immunoresearch Laboratories, West Grove, PA, USA), anti-MAdCAM-1-Alexa Fluor 647 (MECA-367, eBioscience), biotinylated peanut agglutinin (Vector Laboratories, Peterborough, UK), anti-VCAM-1 FITC (clone 429, BD Pharmingen), anti-VCAM-1 biotin (clone M/K-2, Southern Biotech) and anti-podoplanin (8.1.1, kind gift from A. Farr). Anti-CD3ɛ Ab were detected using goat anti-hamster IgG-Cy5 (Jackson). MAdCAM-1 was directly conjugated using the Alexa Fluor 647 Monoclonal Antibody Labeling Kit (Invitrogen). Apart from IgD, FITC-conjugated antibodies were detected using rabbit anti-FITC (Sigma) then goat anti-rabbit IgG-FITC (Jackson). Anti-CCL21 and CXCL13 Ab were detected using donkey anti-goat IgG-Cy3 (Stratech Scientific, Newmarket, UK) or donkey anti-goat FITC (Jackson), rabbit anti-FITC then goat anti-rabbit IgG-FITC. Anti-podoplanin antibodies were detected using goat anti-hamster biotin (Cambridge Biosciences, Cambridge, UK). Biotinylated antibodies were detected using Streptavidin-Alexa Fluor 488, Streptavidin-Alexa Fluor 555 or Streptavidin-Alexa Fluor 647 (all Molecular Probes). Sections were mounted using Vectashield mounting medium (Vector Laboratories).

### Image acquisition and analysis of confocal images

Confocal images were acquired using a Zeiss LSM 510 laser scanning confocal head with a Zeiss Axio Imager Z1 microscope. Digital images were recorded in four separately scanned channels with no overlap in detection of emissions from the respective fluorochromes. Confocal micrographs were stored as digital arrays of 2048×2048 pixels with 8-bit sensitivity; detectors were routinely set so that intensities in each channel spanned the 0–255 scale optimally. Images were analyzed using LSM 510 (Zeiss) as described by Bekiaris *et al*. 11.

### Generation of bone marrow chimeras

Total BM was collected from the hind legs of RAG-B6 mice and 10 million cells were injected i.v. into CD3ɛ mice previously irradiated with 800 rads. Recipient mice were maintained on oral antibiotics for 7 days prior to the irradiation and for the duration of the experiment.

### Flow cytometry

Splenic tissue was teased apart using forceps, followed by red blood cell lysis. Cells were then suspended in PBS/0.1% fetal calf serum and stained with Ab recognizing CD45.1-FITC (eBioscience), and combinations of CD3ɛ-PerCP (145-2C11, BD Biosciences), CD4-PE (L3T4), CD8-APC (Ly-2), CD11c-biotin (N418) and CD19-PE (6D5) (all eBioscience). Biotinylated CD11c was detected using Streptavidin-PE (BD Biosciences). Data were acquired using a Becton-Dickinson LSR FACS machine and analyzed using FlowJo software. For analysis of splenic LTi-like cells, tissue was digested with Collagenase D (Roche Diagnostics, 1 mg/mL), then forced through a nylon filter, before red blood cell lysis and overnight culture. After resuspending in PBS/0.1% fetal calf serum cells from grafts were stained with CD45.1-FITC (A20), CD4-APC (L3T4), B220-PB (RA3-6B2), CD11c-PE (N418) (all eBioscience) and c-kit-biotin (CD117, 2B8) or OX40L-biotin (RM134L) followed by Streptavidin-PeCy5.5 (all BD Biosciences). Cells from BM chimeras were stained for CD45.2-FITC (104, BD Biosciences), CD4-APC, B220-PE (BD Biosciences), CD11c-PE, and c-kit-biotin or OX40L-biotin as above or alternatively for CD45.2-FITC, CD4-APC, CD11c-PE and B220-biotin (BD Biosciences) followed by Streptavidin-PeCy5.5. DAPI (final concentration 0.25 μg/mL) was added to samples prior to acquisition. Data were acquired using a CYAN ADP flow cytometer and analyzed using Summit software (Beckman Coulter, High Wycombe, UK).

### Statistical analysis

All statistical analyses were performed with the non-parametrical Mann–Whitney *U*-test using StatView 5.0 (*p*<0.05 is considered significant).

## Abbreviations

ED15: embryonic day 15
LT: lympotoxin
LTi: lymphoid tissue inducer
PP: Peyer's patches
