# Supplementary material for: Transplantation of embryonic spleen tissue reveals a role for adult non-lymphoid cells in initiating lymphoid tissue organization
Source: Eur J Immunol. 2009 Jan;39(1):280–9. doi: 10.1002/eji.200838724 (PMC3149129; doi:10.1002/eji.200838724)

Supplementary Figure 1

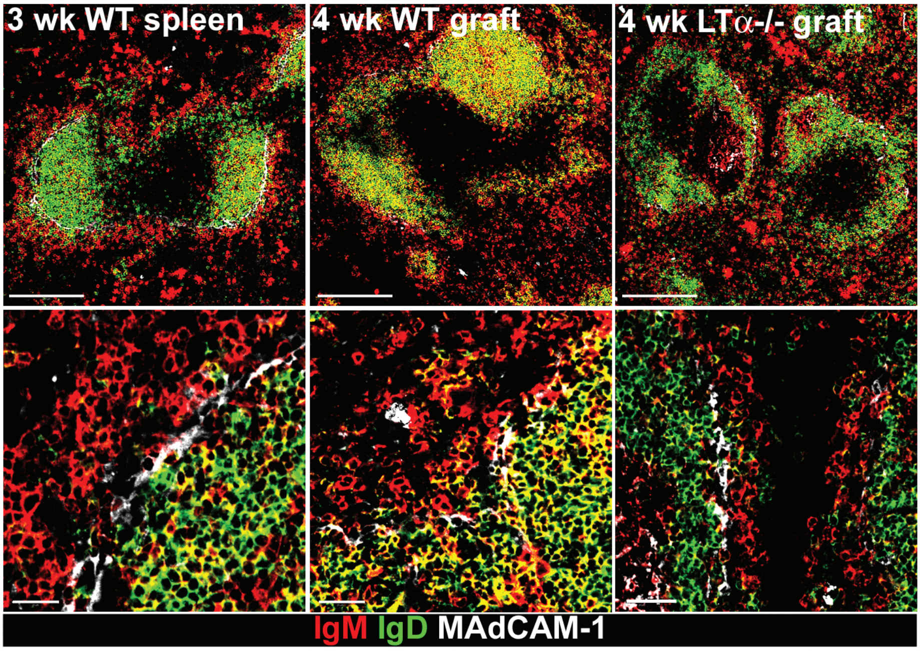

Supplementary Figure 2

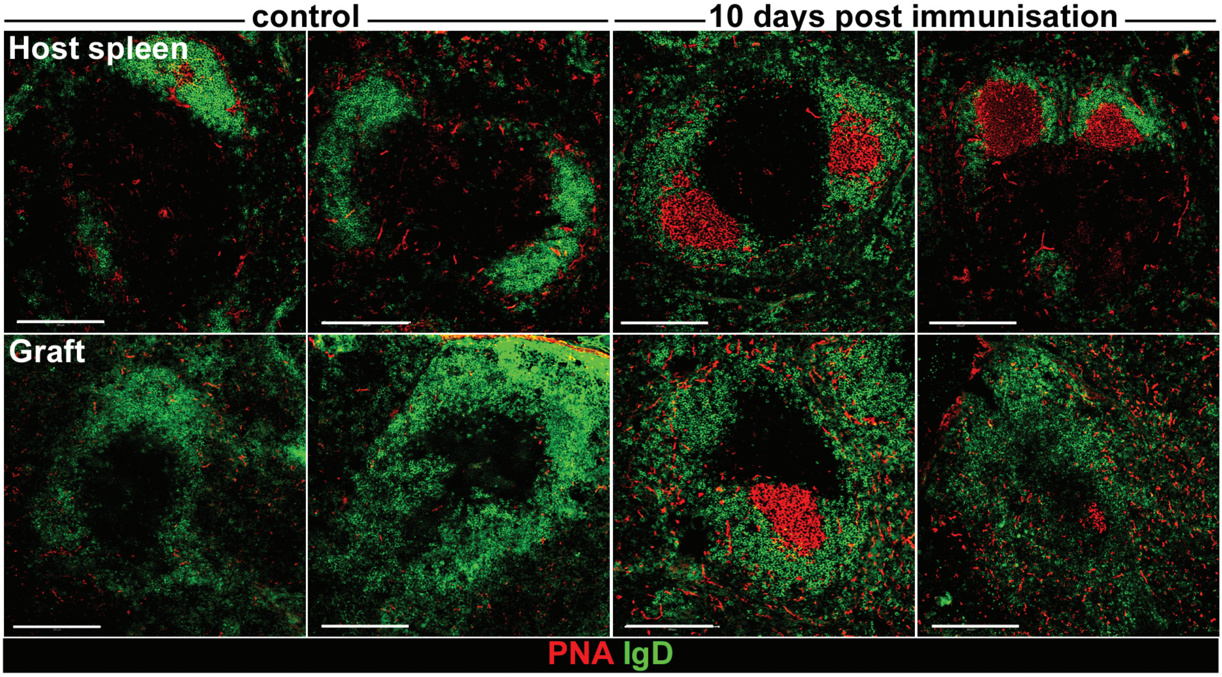

Supplementary Figure 3

LTα-/- graft into LTα-/- host

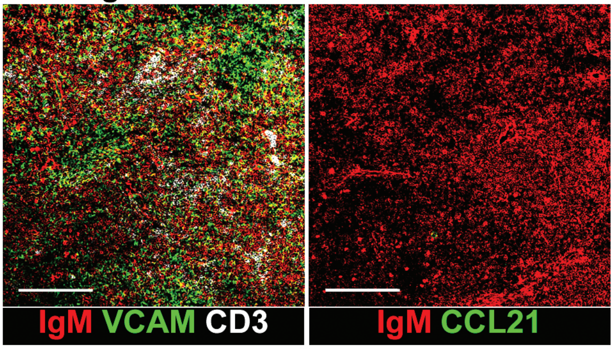

Supplement: Supplementary file 1 [file eji0039-0280-SD1.pdf]
